# Combining partial liver resection and local ablation of liver tumours: a preliminary Dutch experience

**DOI:** 10.1186/1477-7819-4-46

**Published:** 2006-07-17

**Authors:** Bram Fioole, Maarten C Jansen, Frederieke H van Duijnhoven, Richard van Hillegersberg, Thomas M van Gulik, Inne HM Borel Rinkes

**Affiliations:** 1 Department of Surgery, University Medical Centre Utrecht, Utrecht, The Netherlands; 2 Department of Surgery, Academic Medical Centre, Amsterdam, The Netherlands; 3 Department of Surgery, Leiden University Medical Centre, Leiden, The Netherlands

## Abstract

**Background:**

The combination of partial liver resection and radiofrequency ablation (RFA) is a novel concept in the treatment of unresectable liver malignancies. The aim of this study is to evaluate the results of this combined strategy in the Netherlands.

**Methods:**

Thirty-five patients treated with a combination of partial liver resection and RFA were identified from a prospectively registered pooled multicentre database. All patients were operated between June 1999 and November 2003 in 8 medical centres in the Netherlands. Main outcome parameters were morbidity, mortality, local success rate, and survival.

**Results:**

Thirty-seven operations were performed in 35 patients. The group consisted of 20 male and 15 female patients with a median age of 59 years (range 41–76). Seventy-six lesions were resected and RFA was performed to ablate 82 unresectable liver tumours. Twelve patients developed a total of 24 complications, resulting in an overall perioperative morbidity rate of 32%. In two patients major complications resulted in postoperative death (postoperative mortality rate 5.4%). Local success rate after RFA was 88% and the estimated 1-, 2- and 3-year overall survival rates were 84%, 70% and 43%, respectively.

**Conclusion:**

This strategy should only be performed following strict patient selection and within the context of prospective clinical trials.

## Background

Partial liver resection is a well established treatment for primary and secondary malignancies of the liver [1,2]. In a selected population this is the sole potentially curative treatment and is associated with reported 5-year survival rates between 18 and 38% [3-5]. However, the great majority of patients are not amenable to partial liver resection. Reasons to exclude patients for resection are extrahepatic disease, tumour location, inadequate functional hepatic reserve or proximity to major vascular structures.

In recent years, locally ablative therapies have been used as an alternative treatment for unresectable liver tumours. The most widely used technique is radiofrequency ablation (RFA). With this method a radiofrequent current is applied through a needle electrode inserted directly into the tumour, with the aim of destroying all malignant cells. This technique is considered safe and efficient [6,7]. RFA is a low risk procedure, with mortality rates of about 0.5 % and complication rates between 6.9 and 9.8 % [8-11]. However, especially following percutaneous RFA, local recurrence rates up to 40% are reported in large series [12-14]. Tumour recurrences at other sites are common (28–70%), probably caused by negative patient selection [12,13,15]. The overall outcome after RFA is difficult to interpret, since results vary widely and follow up data are immature.

Partial liver resection combined with RFA is a novel approach used in patients with otherwise unresectable lesions. Following resection, the remaining unresectable liver tumours can be ablated, while preserving adequate hepatic reserve. Although RFA has not yet proven to be potentially curative, more patients become candidates for surgical treatment by combining techniques with the purpose of complete clearance of intrahepatic tumour.

The objective of this study was to determine the results of hepatic resection combined with RFA in the Netherlands with emphasis on perioperative morbidity and mortality, recurrence rates and survival. The results were compared with those reported in the literature.

## Methods

Patients were selected from our prospectively recorded multicentre database. Thirty-five patients were identified who had been treated with a combination of partial liver resection and RFA. All patients were operated between June 1999 and November 2003 in 8 medical centres in the Netherlands.

Preoperative work up consisted of a baseline history and physical examination, serum laboratory tests, chest radiograph or chest computed tomography (CT) when indicated, liver ultrasonography (US) or abdominopelvic imaging with CT, magnetic resonance imaging (MRI) or positron emission tomography (PET). The volume of the remnant liver was assessed preoperatively. At least 30–35% of functional liver parenchyma should be preserved. Patients were considered unresectable based on advanced bilobar tumour distribution, inadequate functional hepatic reserve or proximity to major vascular structures. In all patients complete tumour clearance was not possible without the addition of RFA.

All patients underwent partial liver resection and RFA during laparotomy by an experienced liver surgeon. Intraoperative US was performed prior to the resection and RFA to document and target the lesions. During RFA, US was used to guide placement of the RF needle into the lesion and to monitor the effect of the treatment. Routinely, ultrasonic dissection technique was used to perform partial liver resection. Three different RF systems were used in the 8 medical centres (Radionics, Radiotherapeutics and RITA Medical Systems, respectively). Specific RFA protocols, designed by each of the three manufacturers, were used for each system according to manufacturer recommendations. A safety margin of 0.5–1.0 cm was aimed for in surgery as well as in RFA.

Medical records were reviewed for demographics, prior treatment, co-morbidity, tumour histology, number, size and distribution of tumours, type and extent of resection, application of Pringle manoeuvre, operative blood loss and length of hospital stay. Co-morbidity was defined as disease associated with an increased risk for operative complications.

Main outcome parameters were morbidity, mortality, recurrence and survival. Complications were considered major, when 1) life threatening if left untreated, 2) leading to substantial disability, or 3) resulting in hospital readmission or substantially lengthened hospital stay [16]. All other complications were considered minor. All complications following a procedure were registered. When complications were clearly caused exclusively by the ablation procedure they were considered RFA-related. Liver failure was defined as a progressive increase of serum bilirubin and the prothrombin time [17]. Postoperative mortality was defined as in-hospital mortality. Follow up was obtained by multiphasic CT or MRI at 3–4 month intervals following therapy. Recurrence was defined as newly developed tumours in untreated liver segments. Local tumour progression was defined as progression of residual unablated tumour or recurrence of tumour in the RFA lesion. Survival was estimated using Kaplan-Meier analysis.

## Results

Thirty-seven operations were performed in 35 patients. The group consisted of 20 male and 15 female patients with a median age of 59 years (range 41–76).

The indication for treatment was colorectal metastases in 27 patients (77%), other liver metastases in 6 and hepatocellular carcinoma (HCC) in 2 patients (table 1). The main indications for RFA was tumour proximity to major vascular structures and bilobular distribution (table 2). Nine patients had been treated for disseminated disease prior to the combined partial liver resection and RFA. Six patients had received systemic chemotherapy, one patient had previously undergone partial liver resection, one RFA and one chemotherapy via hepatic artery infusion. Thirteen patients (37%) were known to have relevant co-morbidity, including severe pulmonary and cardiac disorders, cirrhosis and diabetes mellitus. Co-morbidity was not associated with increased postoperative morbidity or mortality. Most patients were evaluated by preoperative multiphasic CT (n = 34), but also by MRI (n = 5) and PET scanning (n = 1). All but one patient were operated with curative intent, but none could be treated with partial liver resection alone to remove all lesions while leaving sufficient vital hepatic parenchyma.

**Table 1:** Tumour histology in 37 combined partial liver resections and RFA in 35 patients

Metastases	
Colorectal*	27
Carcinoid	2
Melanoma	1
Ovary carcinoma	1
Breast carcinoma	1
Grawitz	1

Hepatocellular carcinoma	2

*Two patients were treated twice with RFA and partial liver resection

**Table 2:** Indication for RFA in 37 combined partial liver resections and RFA in 35 patients

Tumour proximity to major vascular structures	13
Bilobular tumour distribution	12
Recurrence after surgical treatment	4
Extrahepatic disease	3
Inadequate functional hepatic reserve	3
Cirrhosis	2
Total	37

*Two patients were treated twice with RFA and partial liver resection

One hundred fifty-eight primary and metastatic liver tumours were treated by either partial liver resection or RFA. Seventy-six lesions were resected with a mean (± SD) of 2.1 ± 1.8 lesions per patient per laparotomy (range 1–9). Seven patients (19%) underwent a major resection (table 3). RFA was performed to ablate 82 unresectable liver tumours, with a mean of 2.2 ± 1.5 lesions per patient per laparotomy (range 1–7), and the median diameter of the treated lesions was 19 mm (range 5–50 mm). The Pringle manoeuvre was used in 4 patients. These were all cases in which a tumour was located near major vessels and vascular occlusion was used to reduce the heat sink effect. Median blood loss was 550 ml (range 150–3500). Median operating time was 240 minutes (range 120–600).

**Table 3:** Largest resection in 37 patients treated with combined partial liver resection and RFA

Major resection	
Extended left hemihepatectomy	1
Right hemihepatectomy	2
Segmentectomy	
4 segments	1
3 segments	3

Minor resection	

Segmentectomy	
2 segments	5
1 segment	14
Metastasectomy	11

Total	37

Median hospital stay was 8 days (range 4–25). Twelve patients developed a total of 24 complications, resulting in an overall perioperative morbidity rate of 32%. Minor complications included respiratory tract infection, pleural effusion, thrombosis, cholangitis, urinary tract infection and wound infection (table 4). Fourteen complications in 6 patients were considered major (major complication rate 16%), including liver failure, biliary stricture, hepatic abscess, necrotizing pancreatitis, peritonitis, postoperative haemorrhage and pulmonary embolus. Seven complications in 6 patients were considered solely RFA-related (RFA-related morbidity 16%), including biliary stricture, hepatic abscess and vascular damage. Three patients developed biliary stricture following local ablation. In 1 patient a metastasis treated by RFA was located in segment VIII, in the other 2 patients colorectal metastases in segment IV were treated by local ablation. In all 3 patients local ablation was performed centrally in the liver, resulting in a stricture of the left or right hepatic duct. These patients developed jaundice with peak bilirubin levels ranging from 74 to 940 μmol/L. In all of these cases one or more ERCP procedures were performed for stenting of the biliary tract. Three patients developed hepatic abscesses, 2 of which were located at the site of ablation and, thus considered RFA-related. Treatment consisted of percutaneous drainage (n = 1) or drainage via laparotomy (n = 2). In one patient right portal vein thrombosis near the RFA lesion was diagnosed during analysis for severe ascites. In a second patient minor haemorrhage was observed near the RFA-lesion as result of hepatic artery damage (table 4).

**Table 4:** Twenty-four complications in 12 patients after combined partial liver resection and RFA

*Major*	Number	RFA related
Liver failure	3	0
Biliary stricture	3	3
Hepatic abscess	3	2
Peritoneal infection (S. Aureus)	2	0
Necrotising pancreatitis	1	0
Intrahepatic haematoma	1	0
Pulmonary embolus	1	0

*Minor*		

Respiratory tract infection	2	0
Pleural effusion	2	0
Hepatic vein thrombosis	1	0
Hepatic artery damage	1	1
Cholangitis	1	0
Urine tract infection	1	0
Portal vein thrombosis	1	1
Wound infection	1	0

Total	24	7

*Two patients were treated twice with RFA and partial liver resection

In two patients major complications resulted in postoperative death (postoperative mortality rate 5.4%). Both patients developed liver failure and died in spite of maximum supportive therapy. The first patient had Child-Pugh A cirrhosis as a result of hepatitis C and did not tolerate resection of segment IV and RFA of 2 hepatocellular carcinomas in segments VII and VIII. Postoperatively she developed an intrahepatic haematoma in the left lateral liver lobe due to persisting bleeding from wound tissue. This resulted in a hypovolumic shock requiring multiple blood transfusions, and liver failure. She developed an infected peritonitis (*Staphylococcal aureus *and *Difteroids*) and underwent explorative laparotomy during which necrotic liver parenchyma was resected. This did, however, not result in a clinical improvement and she eventually died due to fatal liver failure. The second patient, with normal preoperative liver function, underwent right hemihepatectomy with 9 colorectal metastases and RFA of a metastasis with a diameter of 20 mm in segment II/III. He was diagnosed with a liver abscess, liver failure and abdominal peritonitis. During relaparotomy a paracolic haematoma infected with Staphylococcal aureus and Streptococcus was removed. Autopsy revealed that death was due to septic shock secondary to necrotizing pancreatitis. In the middle hepatic vein partial thrombosis was observed. This was not related to the RFA procedure, since this metastasis was located deep in segment II/III. The patient did not have portal vein thrombosis.

Median follow-up in the entire study group was 13 months (range 1–46). The estimated 1-, 2- and 3-year overall survival rates were 84%, 70% and 43%, respectively. Estimated disease-free 1-, 2- and 3-year survival rates were 57%, 38% and 20%, respectively. Irradical resection was observed in 5 patients. In 4 patients the resection appeared irradical at histopathological examination, while in 1 patient optimal extrahepatic cytoreduction of stage IV ovarian carcinoma could not be achieved. During follow-up local tumour progression was observed in 10 RFA lesions (in 8 patients) as indicator of local treatment failure, resulting in a local success rate of 88%. All tumours were colorectal metastases. Four of these tumours were larger than 3 cm. Four other tumours were smaller, but were localized in the dome or centrally in the liver (segment IVA, VI or VII). For the remaining 2 tumours no explanation for incomplete ablation could be found. Median time to local tumour progression was 5 months. Seventeen patients (46%) developed hepatic recurrence, while 15 patients (41%) developed extrahepatic disease. The median number of liver tumours treated by local ablation per operation was 4 (range 2–10). In patients with ≥ 4 tumours recurrence was 58%, while in patients with < 4 tumours recurrence was 33%, respectively (p = 0.12).

One single patient was treated for ovarian carcinoma stage IV by multimodality therapy including chemotherapy (Taxol), cytoreductive surgery and combined partial liver resection and RFA. Optimal extrahepatic cytoreduction (< 1 cm residual disease) could not be achieved and after 4 months recurrence in the liver was observed. Two other patients were also known to have extrahepatic disease (table 2). In the first patient two colorectal lung metastases were additionally removed. Three months after resection and RFA hepatic recurrence was observed and after 6 months local tumour progression and new extrahepatic lesions were diagnosed. The second patient underwent an additional metastasectomy of an omental metastasis of a Grawitz tumour. After 15 months of follow-up the patient is still free of disease.

## Discussion

Several series have been published on combined resection and RFA [7,18-22]. However, all of these involved mono-institutional experience of large cancer centres. We report the results from a Dutch multi-institutional database, involving 8 hospitals, including 1 cancer centre. Although all resections and RFA procedures were performed by experienced liver surgeons, this series may reflect daily practice more accurately.

In the current series, we observed a morbidity rate of 32% (major complication rate 16%) and a mortality rate of 5.4%. In the same period 87 patients were solely treated by RFA in the hospitals involved in this study. In this group the mortality rate after RFA was 0% and the RFA related morbidity was 9.8% [11]. In a series from the University Medical Centre Utrecht the mortality and morbidity rate, following partial liver resection were 5.8% and 23% respectively. The 5-year survival rate after liver resection for colorectal metastases was 38%. [23]. Better figures were reported by Pawlik *et al*, with a postoperative complication rate of 19.8% and an overall mortality rate of 2.3% in 172 patients treated with partial liver resection and RFA, among which 54% were major hepatectomies [7], while numbers similar to ours were found by Mulier *et al*, reporting a complication rate of 31.8 % and a mortality rate of 4.5% in patients treated with open RFA in combination with cryotherapy, partial hepatic resection or extrahepatic resection [8]. Elias *et al*, did not observe any postoperative deaths in 63 patients and found a morbidity rate of 27% [21]. These series show that morbidity and mortality rates of combined partial liver resection and RFA are comparable with those of partial liver resection alone [1-3].

In large series the 5-year survival is 36–39% after partial liver resection alone as treatment for colorectal liver metastasis, which is still considered the gold standard [3,4,24]. Although few series have been published describing long-term results after RFA, the results appear promising. Solbiati *et al*, report a 3-year survival rate of 46% and Gillams *et al*, report a 5-year survival rate of 30% [12,13,25]. Abdalla *et al*, compared RFA only, RFA + resection, resection only and chemotherapy retrospectively in a large series (n = 418) [20]. They described that the combination of partial liver resection and RFA in 101 patients provided only modest survival benefit over chemotherapy alone and was not different than following RFA only in patients with colorectal liver metastases. Survival after resection only was significantly better than all other treatment modalities. In our series 3-year overall survival was 43%, while the disease-free survival was 20%. Due to the number of patients with various diseases in our series, conclusions with regard to oncological outcome cannot be drawn from the present study.

Patient selection strongly improves the results in liver surgery. Since substantial morbidity and mortality are introduced, patients should be selected rigorously when treated with combined resection and RFA. In our series, 1 patient died after right hepatectomy and controlateral RFA of a colorectal metastasis measuring 20 mm. For patients treated by combined major resection and RFA, percutaneous RFA combined with portal vein embolization followed by hepatectomy one month later should ideally be considered [26].

With regard to long term outcome the following aspects should be taken into consideration. The total number of tumours has been identified as a factor affecting time to recurrence after resection alone [27-29] or combined resection and RFA [7,20]. In this series, there was a trend for an increased recurrence rate in patients treated for ≥ 4 tumours (p = 0.12). Since tumour diameter is also a risk factor for local recurrence after RFA, only small tumours should be treated by RFA when combined with resection [12,30]. A cut off point of 30 mm diameter has been proposed [21]. In general percutaneous RFA should not be performed, since this approach results in increased local recurrence [30]. A fourth risk factor affecting long term survival is extrahepatic disease [28,31]. Although a 5-year survival rate of 28% after "R0" resection of extrahepatic disease in conjunction with partial hepatectomy for colorectal liver metastases has been published, this intended "R0" resection could not be performed in 50% of operated patients [32]. Therefore, in our opinion extrahepatic disease is a relative contraindication for combined resection and RFA. Only in patients with intraoperative potential for "R0" resection and simple extrahepatic disease after meticulous exploration, combined resection and RFA should be considered. We have treated only 3 patients with extrahepatic disease in our series with variable outcome. A fifth factor influencing survival might be chemotherapy. Neoadjuvant chemotherapy allows 10–15% of patients with unresectable colorectal liver metastases to be rescued by liver surgery [33]. In a series of Elias, et al the strategy of combined partial liver resection and RFA with perioperative chemotherapy appeared favourable by doubling the median survival (from 18 to 36 months), when compared to similar unresectable colorectal patients treated by chemotherapy alone [21]. RFA followed by chemotherapy seems feasible, but no randomised studies on its additive value are available at present [21,34].

## Conclusion

Combining partial liver resection and RFA augments the number of patients who can be treated surgically. But this strategy should only be performed following strict patient selection and within the context of prospective clinical trials.

## Competing interests

The author(s) declare that they have no competing interests.

## Authors' contributions

**BF **designed the study, carried out the data acquisition and drafted the manuscript. **MJ **and **FvD **carried out the data acquisition and participated in drafting and revising the manuscript. **RvH **and **TvG **participated in the design of the study and in drafting and revising the manuscript. **IBR **conceived of the study and coordinated the draft and revision of the manuscript. All authors read and approved the final manuscript.
